# Residual passive anterior tibial subluxation following anterior cruciate ligament reconstruction using hamstring tendon autografts: A longitudinal magnetic resonance imaging study with a minimum 2‐year follow‐up

**DOI:** 10.1002/jeo2.70793

**Published:** 2026-05-25

**Authors:** Yi‐tian Gao, Wei‐li Shi, Zhi‐yu Zhang, Wen‐bin Bai, Zi‐hao Zhou, Yu‐ping Yang, Yong Ma, Xi Gong, Cheng Wang, Jian‐quan Wang

**Affiliations:** ^1^ Department of Sports Medicine Peking University Third Hospital, Institute of Sports Medicine of Peking University Beijing China; ^2^ Beijing Key Laboratory of Research and Translation for Drugs and Medical Devices in Precision Diagnosis and Treatment of Sports Injuries Beijing China; ^3^ Engineering Research Center of Sports Trauma Treatment Technology and Devices, Ministry of Education Beijing China

**Keywords:** anterior cruciate ligament, anterior tibial subluxation, anterolateral ligament, longitudinal change, meniscal injury

## Abstract

**Purpose:**

To track the 24‐month longitudinal changes in tibiofemoral alignment characterised by passive anterior tibial subluxation (PATS) following anterior cruciate ligament reconstruction (ACLR), and to investigate associated factors.

**Methods:**

Fifty‐one patients who underwent primary ACLR using hamstring tendon autografts between March 2021 and February 2022 were enrolled. Clinical and magnetic resonance imaging (MRI) evaluations were performed at baseline (within 3 days preoperatively) and at 6‐, 12‐ and 24‐month follow‐ups to examine the graft integrity and tibiofemoral alignment. Lateral PATS (L‐PATS), medial PATS (M‐PATS), global PATS (G‐PATS) and rotational PATS (R‐PATS) were measured on serial MRI. The repeated‐measures one‐way analysis of variance was applied to test the longitudinal changes in PATS. Univariable and multivariable linear regression analyses were performed to identify associations between preoperative and postoperative PATS, adjusting for a priori‐defined covariates including time from injury to surgery, tibial slopes, meniscal injuries and anterolateral ligament (ALL) abnormality.

**Results:**

The graft integrity was clinically and radiographically confirmed in all patients at the 24‐month follow‐up. However, serial MRI revealed significant increases in L‐PATS, M‐PATS and G‐PATS (all *p* < 0.001) following the primary ACLR. The increases in L‐PATS (1.5 mm, 95%CI [0.6,2.5], *p* < 0.001), M‐PATS (1.2 mm, 95%CI [0.5,1.9], *p* < 0.001) and G‐PATS (1.4 mm, 95%CI [0.6,2.1], *p* < 0.001) from baseline became the most prominent at 12 months and remained stable thereafter. Strong correlations were identified between preoperative and 24‐month postoperative values for L‐PATS (*β* = 0.60, *p* < 0.001), M‐PATS (*β* = 0.43, *p* < 0.001), G‐PATS (*β* = 0.48, *p* < 0.001) and R‐PATS (*β* = 0.70, *p* < 0.001). ALL abnormality was associated with increased L‐PATS (*β* = 1.85, *p* = 0.008) and G‐PATS (*β* = 1.31, *p* = 0.009), while medial meniscal injury was associated with increased M‐PATS (*β* = 1.12, *p* = 0.036) and G‐PATS (*β* = 0.96, *p* = 0.048) measured at 24 months postoperatively.

**Conclusions:**

Residual tibiofemoral malalignment characterised by increased L‐PATS, M‐PATS and G‐PATS persists following ACLR using hamstring tendon autografts. Excessive preoperative PATS, ALL abnormality and medial meniscal injury are associated with increased postoperative PATS.

**Level of Evidence:**

Level IV.

## Graphical abstract

Residual tibiofemoral malalignment characterised by passive anterior tibial subluxation can persist following anterior cruciate ligament reconstruction.

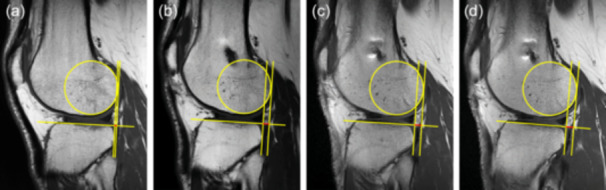

**Abbreviations**
ACL: anterior cruciate ligament
ACLR: anterior cruciate ligament reconstruction
ALL: anterolateral ligament
ANOVA: analysis of variance
BMI: body mass index
CI: confidence interval
G‐PATS: global passive anterior tibial subluxation
ICC: intraclass correlation coefficient
IKDC: International Knee Documentation Committee
LM: lateral meniscus
LTS: lateral tibial slope
L‐PATS: lateral passive anterior tibial subluxation
MM: medial meniscus
MRI: magnetic resonance imaging
MTS: medial tibial slope
M‐PATS: medial passive anterior tibial subluxation
PATS: passive anterior tibial subluxation
R‐PATS: rotational passive anterior tibial subluxation
SD: standard deviation
TFIS: time from injury to surgery.

## INTRODUCTION

Anterior cruciate ligament (ACL) injuries commonly lead to altered tibiofemoral alignment and abnormal knee kinematics [7, 19, 32], thereby disturbing the physiological loading pattern and predisposing injured knees to an increased risk of osteoarthritis [3]. Despite the generally satisfactory clinical outcomes achieved through anterior cruciate ligament reconstruction (ACLR), evidence suggests that the tibiofemoral alignment, whether measured dynamically by gait analysis or statically by radiography [1, 2], is not fully restored by isolated intraarticular reconstructions. It has been reported that the tibiofemoral malalignment caused by an ACL injury may persist even when the knee stability is restored [1, 2, 25, 37], raising concerns regarding the long‐term outcomes of ACL‐reconstructed knees [30].

Widely adopted as an accurate parameter to quantify the tibiofemoral malalignment, passive anterior tibial subluxation (PATS) measured on magnetic resonance imaging (MRI) has been extensively studied in ACL‐deficient knees [19, 32]. The presence of excessive PATS has been validated in both primary and secondary ACL‐injured knees [6, 19, 32], demonstrating associations with osseous morphologies and the integrity of secondary stabilisers [19, 27, 39]. Although the precise cause of this phenomenon has not been entirely clarified, recent studies have revealed the clinical relevance of increased preoperative PATS, highlighting its associations with inferior knee stability [15, 17, 36], poor functional outcomes [28] and compromised graft survivorship [6, 19, 32]. However, whether and to what extent PATS can be reduced by ACLR remains controversial.

The restoration of PATS is observed when measured immediately following ACLR [20, 33]. However, multiple studies have reported that PATS may persist or deteriorate during follow‐up despite clinically successful reconstructions and overall improved stability [1, 2, 25, 37], especially in patients demonstrating excessive PATS preoperatively [4, 5, 26]. These conflicting findings might be attributed to a lack of longitudinal observations, as a single measurement of PATS is insufficient to characterise tibiofemoral alignment in the long term [6, 18]. Knowledge regarding the longitudinal change and associated factors of postoperative PATS may help identify patients at higher risk of residual tibiofemoral malalignment, and guide the implementation of the individualised surgical interventions to mitigate the associated detrimental effects [16, 29, 35].

The aims of the present study were (1) to track the 24‐month longitudinal changes in PATS following ACLR and (2) to investigate the factors associated with postoperative PATS. It was hypothesised that (1) residual PATS would persist following ACLR and (2) postoperative PATS would be associated with preoperative PATS and secondary stabiliser injuries.

## METHODS

### Study design and patient selection

This study was approved by the institutional review board (M2020384) and registered (ChiCTR2100043440) before enrollment. Informed consent forms were signed by all participants voluntarily. Between March 2021 and February 2022, consecutive patients diagnosed with ACL injuries and treated by a single surgical team at a tertiary medical centre were screened for eligibility. Patients were included based on the following criteria: (1) clinically diagnosed with primary ACL ruptures and indicated for ACLR; (2) aged 18–50 years; (3) able and willing to be followed up for 2 years postoperatively. Patients were excluded for meeting at least one of the following criteria: (1) previous injury to either knee; (2) multi‐ligamentous injury requiring surgery; (3) advanced knee osteoarthritis (Kellgren‐Lawrence grade ≥3); (4) severe varus/valgus deformity ≥ 5°; (5) total meniscectomy. Patients missing any of the clinical or MRI follow‐ups were further excluded from the final analysis.

### Surgical technique

All patients underwent standardised, isolated, anatomic, single‐bundle ACLR performed by two senior fellowship‐trained surgeons (C.W. and X.G.). Following arthroscopic confirmation of ACL and meniscal status, meniscal repair or partial meniscectomy was performed based on the principle of meniscal preservation. Hamstring tendon autografts were harvested from the ipsilateral knee to form a four‐strand grafts with diameters ranging from 7 to 9 mm. Femoral tunnels were drilled via the anteromedial portal at the anatomic footprint, and tibial tunnels were created using a guide at the native attachment site. An Endobutton (Smith & Nephew) was used for femoral fixation, while an interference screw (Smith & Nephew) and a supplementary staple (Smith & Nephew) were used for tibial fixation with the knee in full extension. Postoperative three‐dimensional computed tomography was routinely performed to confirm the anatomic tunnel placement in all patients [24].

### Rehabilitation protocol

A standardised rehabilitation protocol was initiated under the instruction of therapists on the first postoperative day and monitored at 2, 6 and 12 weeks on an outpatient basis for all patients. Isometric quadriceps, straight‐leg raises, patellar mobilisation and ankle pump exercises were started on the first postoperative day. Range of motion exercises started on postoperative Day 4. Full weight bearing was allowed after 6 weeks unless a meniscal repair dictated otherwise. Patients progressed to running after 3 months and returned to sport‐specific training after 10 to 12 months when muscle strength was above 80% of the unaffected knee.

### Clinical evaluation

Clinical and MRI evaluations were performed at baseline (within 3 days preoperatively) and at 6, 12 and 24 months. Physical examinations, including the anterior drawer test and the Lachman test were manually performed by the same two surgeons (C.W. and X.G.) without anaesthesia to assess graft integrity. The relative displacement of the tibia was estimated by the examiner and graded according to the International Knee Documentation Committee (IKDC) as 0 (0–2 mm), 1 (3–5 mm), 2 (6–10 mm) and 3 (>10 mm) based on the comparison between the injured and healthy knees [8] Patient‐reported outcome measures (PROMs) including the subjective IKDC score [11] and the Lysholm score [34] were assessed by an appropriately trained clinician who was not a member of the surgical team and was blinded to the study design.

### MRI evaluation

MRI evaluations were performed with a GE Discovery MR750 3.0‐T System (GE Healthcare) using a standardised institutional protocol. Patients were arranged to maintain the supine position with the knee fully extended in the neutral position. The extremity was secured in a tight‐fitting eight‐channel knee coil (MedRad) with additional padding to ensure a consistent extremity position [32]. Images were obtained in standardised sequences (T1‐weighted and proton‐density weighted) and planes (sagittal, coronal and axial) with a 3‐mm slice thickness. Measurements were performed on the PACS system (INFINITT Healthcare Co.) independently by two sports fellowship‐trained orthopaedic surgeons (Y.M. and Y.‐P. Y.) with more than 10 years of sports medicine experience, who were blinded to the patient information and examination dates. One of the two surgeons (Y.M.) repeated all measurements after a 4‐week interval to evaluate intra and interobserver reliability.

**Figure 1:**
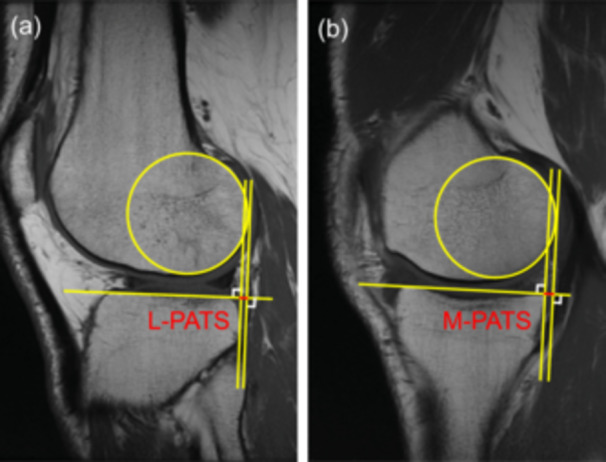
Magnetic resonance images demonstrating the measurement of passive anterior tibial subluxation (PATS) (red segments). (a) Lateral PATS (L‐PATS) was measured on the midsagittal slices of the lateral compartment showing the medial aspect of the fibula head. (b) Medial PATS (M‐PATS) was measured on the midsagittal slices of the medial compartment showing the femoral insertion of the medial gastrocnemius tendon.

*Passive anterior tibial subluxation*. According to the methods described by Tanaka et al. [32], lateral and medial PATS (L‐PATS and M‐PATS) were measured on the midsagittal slices of each compartment demonstrating the medial aspect of the fibula head or the femoral insertion of the medial gastrocnemius tendon, respectively. A best‐fit circle of the posterior femoral condyle was first drawn over the subchondral line. Two lines perpendicular to the tibial plateau were then drawn through the posterior margin of the circle, as well as the tibial plateau. The distance between these two lines was measured in both lateral and medial compartments and reported as L‐PATS and M‐PATS, respectively (Figure 1). Global PATS (G‐PATS) was calculated as the average of L‐PATS and M‐PATS to characterise the overall anteroposterior tibiofemoral relationship [7], while rotational PATS (R‐PATS) was calculated as the difference between L‐PATS and M‐PATS (L‐PATS minus M‐PATS) to characterise the internal rotational tibiofemoral relationship [22].

**Figure 2:**
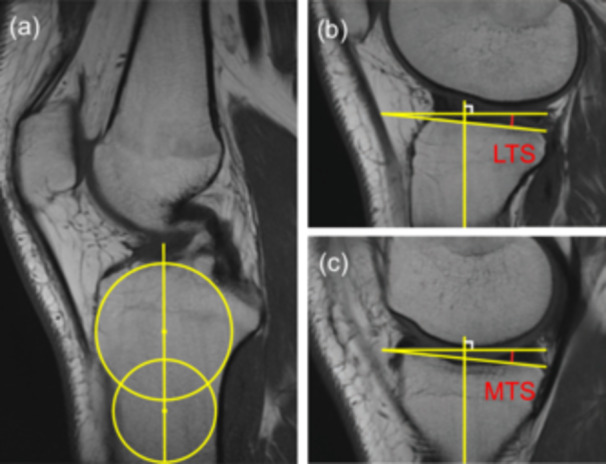
Magnetic resonance images demonstrating the measurement of tibial slopes. (a) The proximal tibial anatomic axis was determined on the central sagittal slice by connecting the centres of two circles placed at the tibial metaphysis. On midsagittal slices of the lateral (b) and medial (c) compartment, lateral tibial slope (LTS) and medial tibial slope (MTS) were measured as complementary angles (red arcs) between the tangential lines of the tibial plateau and the proximal tibial anatomic axis.

*Tibial slopes*. Lateral and medial tibial slopes (LTS and MTS) were measured according to the method described by Hudek et al. [10]. The proximal tibial anatomic axis was determined on the central sagittal slice by connecting the centres of two circles placed within the tibial metaphysis. The complementary angle between the tangential line of the tibial plateau and the proximal tibial anatomic axis was measured in both lateral and medial compartments and documented as LTS and MTS, respectively (Figure 2).

**Figure 3:**
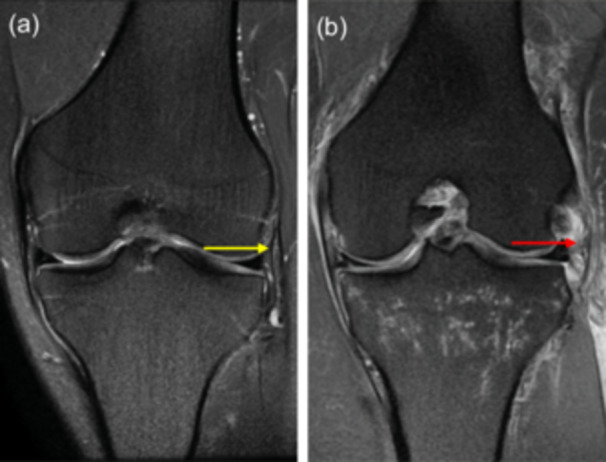
Magnetic resonance images demonstrating the intact (a) (yellow arrow) and abnormal (b) (red arrow) anterolateral ligament.

*Anterolateral ligament (ALL)*. The ALL was evaluated on coronal proton‐density weighted images as a band of tissue deep to the lateral capsule, arising from the lateral femoral epicondyle and inserting into the anterolateral border of the proximal tibia. The status of the ALL was classified as nonvisualised, normal or abnormal based on previously described criteria [9, 19, 36], which have been reported to achieve satisfactory intra and interobserver reliabilities [9]. ALL abnormalities were defined as disrupted continuity, irregular contour or peri‐ligamentous edema, with or without associated bone fragments (Figure 3). In cases of disagreement between the two observers on ALL status, the final decision was made by a senior surgeon (J.‐Q.W.).

### Statistical analysis

The sample size was calculated using G*Power software (Release 3.1.9.7), with 95% power at a significance level of 0.05. Assuming a correlation of 0.5 between the baseline and 24‐month PATS, the calculated sample size was 15 to detect a reduction from 8.1 mm (standard deviation [SD], 1.8 mm) to 5.9 mm (SD, 2.8 mm) in L‐PATS, and 33 to detect a reduction from 5.2 mm (SD, 1.9 mm) to 3.7 mm (SD, 2.9 mm) in M‐PATS postoperatively [16].

Statistical analyses were performed using SPSS (Version 26.0; IBM Corp) and GraphPad Prism (Version 9.5, GraphPad Software). The intraclass correlation coefficient (ICC) was calculated for MRI measurements and categorised as ‘excellent’ (ICC > 0.9), ‘good’ (0.75 < ICC < 0.9), ‘moderate’ (0.5 < ICC < 0.75) or ‘poor’ (ICC < 0.5). Descriptive statistics were reported for patient characteristics. Continuous variables were compared using the paired *t*‐test. Repeated‐measures one‐way analysis of variance (ANOVA) was applied to test the longitudinal changes in PATS, followed by Dunnett's multiple comparisons. After confirming the assumptions, univariable linear regression analysis was performed to identify the associations between the preoperative and postoperative PATS, followed by multivariable linear regression analysis to adjust for a priori‐defined covariates, including time from injury to surgery, tibial slopes, meniscal injuries and ALL abnormality according to previous literature indicating their associations with PATS [19, 27, 38]. Exploratory univariable linear regression analyses were employed to assess the associations between postoperative PATS and PROMs at postoperative 24 months. The level of significance was set at *p* < 0.05.

## RESULTS

### Patient characteristics

**Figure 4:**
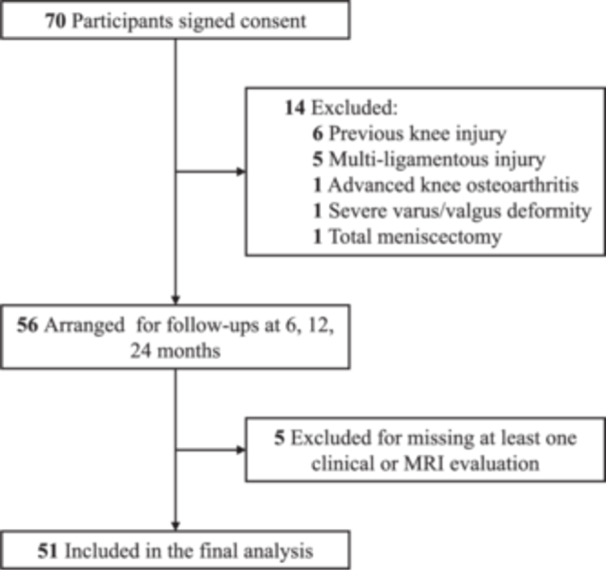
Flow diagram of participants through the study. MRI, magnetic resonance imaging.

Enrollment occurred between March 2021 and February 2022 (Figure 4). Of the 70 patients who signed the informed consent, 14 were excluded based on the exclusion criteria. A total of 51 (91.1%) patients who completed all aspects of the 24‐month follow‐up were included in the final analysis. The cohort comprised 40 male and 11 female participants, with a mean age of 35.7 (SD, 10.8) years. Patient baseline characteristics are summarised in Table 1.

**Table 1:** Patient baseline characteristics.^a^

Characteristic	Total (*n* = 51)
Demographic data	
Age at surgery, years	35.7 ± 10.8
BMI, kg/m^2^	25.4 ± 3.5
Male	40 (78.4)
Beighton score, 0–9	0 [0,2]
Injury characteristic	
Right side	29 (56.9)
Contact injury	4 (7.8)
TFIS, months	4 [1,19]
≤3	24 (47.1)
>3	27 (52.9)
Concomitant injury	
Lateral meniscus	
Intact	27 (52.9)
Injured	24 (47.1)
Repaired	4 (7.8)
Partial meniscectomy	20 (39.2)
Medial meniscus	
Intact	25 (49.0)
Injured	26 (51.0)
Repaired	5 (9.8)
Partial meniscectomy	21 (41.2)
ALL status	
Visualised, normal	17 (33.3)
Visualised, abnormal	28 (54.9)
Nonvisualised	6 (11.8)
Anatomic parameters	
LTS, deg	6.6 ± 3.1
MTS, deg	6.3 ± 2.4

Abbreviations: ALL, anterolateral ligament; BMI, body mass index; LTS, lateral tibial slope; MTS, medial tibial slope; TFIS, time from injury to surgery.

^a^ Data were given as mean ± standard deviation, median [percentile 25th, percentile 75th] or *n* (%).

### Clinical evaluation

There was no fixed flexion deformity or genu recurvatum in any of the included participants preoperatively. A full range of motion at the preinjury level was achieved in all patients postoperatively. No patient presented with a positive anterior drawer test or Lachman test postoperatively, except for one patient who demonstrated a Grade 1A Lachman at the final follow‐up. Significant improvements in the Lysholm score (80.4 ± 8.6 vs. 91.2 ± 8.6, *p* < 0.001) and the IKDC score (74.8 ± 7.3 vs. 89.0 ± 7.9, *p* < 0.001) from baseline were observed at 24 months (Table 2).

**Table 2:** Preoperative and postoperative clinical evaluation.^a^

		Postoperative follow‐ups		
Characteristic	Preop.	6‐month Postop.	12‐month Postop.	24‐month Postop.
Anterior drawer test, grade 0/1/2/3	0/11/39/1	51/0/0/0	51/0/0/0	51/0/0/0
Lachman test, grade 0/1/2/3	0/6/43/2	51/0/0/0	51/0/0/0	50/1/0/0
Lysholm score	80.4 ± 8.6	82.9 ± 12.8	91.4 ± 8.9	91.2 ± 8.6
IKDC score	74.8 ± 7.3	80.4 ± 5.8	89.3 ± 7.4	89.0 ± 7.9

Abbreviation: IKDC, International Knee Documentation Committee.

^a^ Data were given as mean ± standard deviation or No.

### Longitudinal changes in PATS

**Figure 5:**
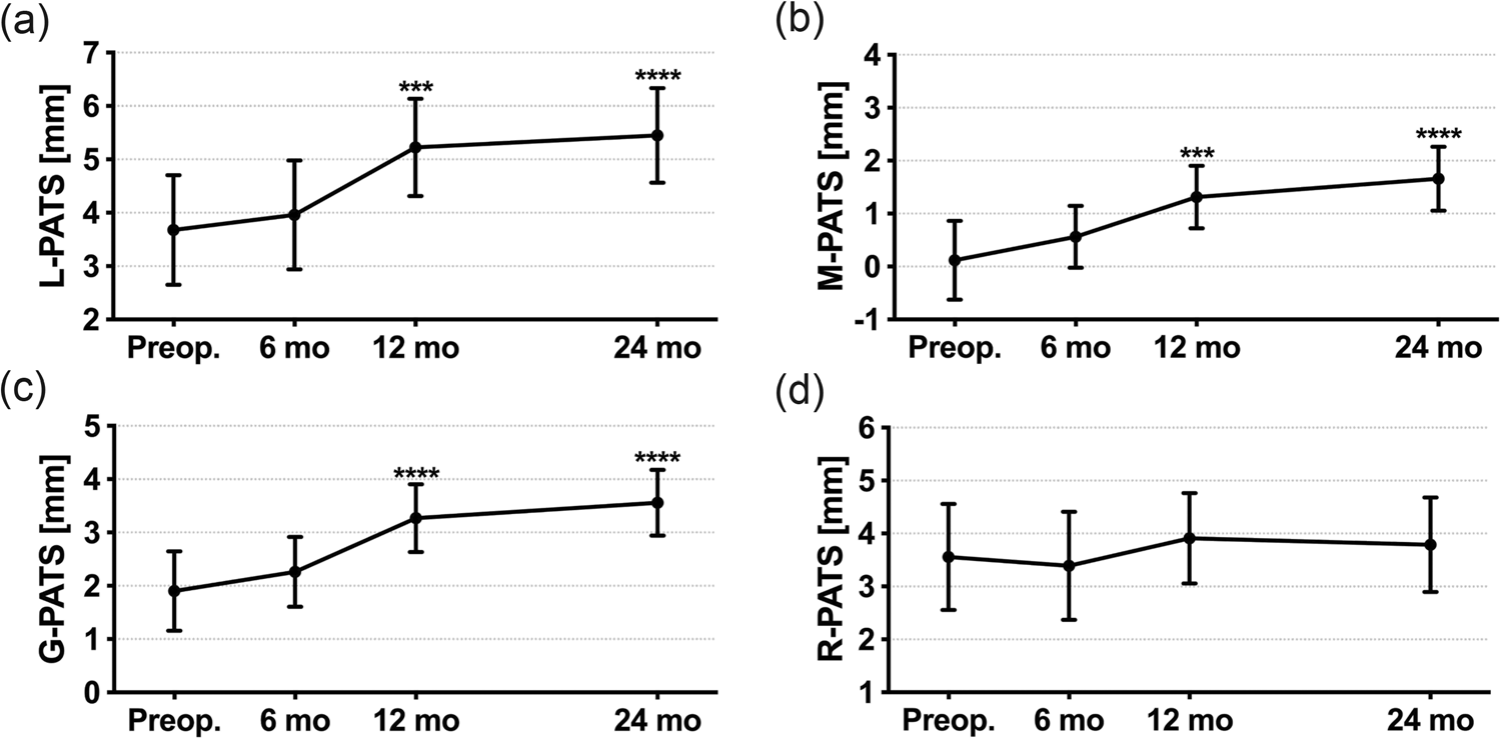
Longitudinal changes in lateral passive anterior tibial subluxation (a), medial passive anterior tibial subluxation (b), global passive anterior tibial subluxation (c) and rotational passive anterior tibial subluxation (d) over time. Data were demonstrated as means and 95% CIs. Multiple comparisons of postoperative measurements with preoperative measurements at each given time point were indicated as follows: *** *p* < .001, **** *p* < .0001. CI, confidence interval.

**Figure 6:**
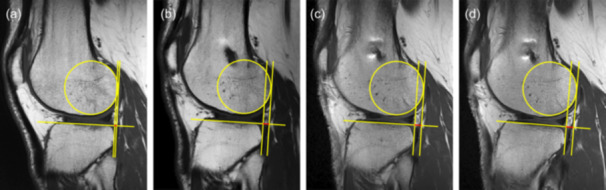
Serial magnetic resonance images demonstrating the longitudinal changes in the lateral passive anterior tibial subluxation (L‐PATS) in a representative case. Measured L‐PATS (red segments) were 1.18 mm at baseline (a), 3.67 mm at 6 months (b), 4.06 mm at 12 months (c) and 4.45 mm at 24 months (d).

Over 24 months, patients who underwent primary ACLR demonstrated significant changes in L‐PATS, M‐PATS and G‐PATS (all *p* < 0.001), but not in R‐PATS (*p* = 0.399) (Table 3). Slight increases in L‐PATS, M‐PATS and G‐PATS were observed at 6 months although not achieving significance (all *p* > 0.05) (Figure 5). By 12 months, significant increases were observed in L‐PATS (1.5 mm, 95% CI [0.6, 2.5]), M‐PATS (1.2 mm, 95% CI [0.5, 1.9]) and G‐PATS (1.4 mm, 95% CI [0.6, 2.1]) compared to baseline measurements (all *p* < 0.001). However, no further progression was noted in L‐PATS (*p* = 0.574), M‐PATS (*p* = 0.230) and G‐PATS (*p* = 0.162) between 12 and 24 months. A representative case of the longitudinal changes in L‐PATS is illustrated in Figure 6. ICC analysis indicated good to excellent intra and interobserver reliabilities for the measurement of L‐PATS (0.917 [95% CI, 0.859–0.952] and 0.872 [95% CI, 0.787–0.925]) and M‐PATS (0.926 [95% CI, 0.874–0.957] and 0.914 [95% CI, 0.854–0.950]).

**Table 3:** Longitudinal changes in PATS over 24 months postoperatively.^a^

		Postoperative follow‐ups			
	Preop.	6‐month Postop.	12‐month Postop.	24‐month Postop.	*p‐* Value^b^
L‐PATS, mm	3.7 ± 3.6	4.0 ± 3.6	5.2 ± 3.2	5.5 ± 3.2	**<0.001**
M‐PATS, mm	0.1 ± 2.7	0.6 ± 2.1	1.3 ± 2.1	1.7 ± 2.2	**<0.001**
G‐PATS, mm	1.9 ± 2.6	2.3 ± 2.3	3.3 ± 2.3	3.6 ± 2.2	**<0.001**
R‐PATS, mm	3.6 ± 3.6	3.4 ± 3.6	3.9 ± 3.0	3.8 ± 3.2	0.399

Abbreviations: ANOVA, analysis of variance; G‐PATS, global passive anterior tibial subluxation; L‐PATS, lateral passive anterior tibial subluxation; M‐PATS, medial passive anterior tibial subluxation; PATS, passive anterior tibial subluxation; R‐PATS, rotational passive anterior tibial subluxation.

^a^ Data were given as mean ± standard deviation.

^b^ *P* Value derived from repeated measures one‐way ANOVA. Bold values indicate statistical significance.

### Relationship between preoperative and postoperative PATS

Univariable linear regression analysis demonstrated strong correlations between preoperative and 24‐month postoperative values for L‐PATS, M‐PATS, G‐PATS and R‐PATS (all *p* < 0.001) (Table 4). After adjustment for a priori‐defined covariates in multivariable regressions, the observed relationships remained significant for L‐PATS (*β* = 0.60, *p* < 0.001), M‐PATS (*β* = 0.43, *p* < 0.001), G‐PATS (*β* = 0.48, *p* < 0.001) and R‐PATS (*β* = 0.70, *p* < 0.001). Moreover, ALL abnormality at the time of primary injury was found to be associated with increased 24‐month L‐PATS (*β* = 1.85, *p* = 0.008) and G‐PATS (*β* = 1.31, *p* = 0.009). Medial meniscus injury was also found to be associated with increased 24‐month M‐PATS (*β* = 1.12, *p* = 0.036) and G‐PATS (*β* = 0.96, *p* = 0.048) (Table 4).

**Table 4:** Univariate and multivariate linear regression analysis of factors associated with the 24‐month PATS.^a^

	24‐montn Postop. L‐PATS		24‐month Postop. M‐PATS		24‐month Postop. G‐PATS		24‐month Postop. R‐PATS	
	*β* [95% CI]	*p‐* Value	*β* [95% CI]	*p‐* Value	*β* [95% CI]	*p‐* Value	*β* [95% CI]	*p‐* Value
Univariable linear regression								
Preop. PATS	0.63 [0.46, 0.80]	**<0.001**	0.53 [0.35, 0.70]	**<0.001**	0.55 [0.37, 0.73]	**<0.001**	0.69 [0.53, 0.85]	**<0.001**
Multivariable linear regression								
Preop. PATS	0.60 [0.42, 0.78]	**<0.001**	0.43 [0.24, 0.63]	**<0.001**	0.48 [0.30, 0.66]	**<0.001**	0.70 [0.51, 0.89]	**<0.001**
TFIS > 3 months	0.51 [−0.83, 1.85]	0.444	0.25 [−0.79, 1.29]	0.633	0.42 [−0.56, 1.39]	0.392	0.44 [−0.92, 1.81]	0.515
ALL abnormality	1.85 [0.52, 3.18]	**0.008**	0.70 [−0.34, 1.73]	0.181	1.31 [0.35, 2.27]	0.**009**	0.87 [−0.52, 2.25]	0.214
LM injury	0.58 [−0.66, 1.81]	0.354	0.36 [−0.58, 1.31]	0.441	0.52 [−0.37, 1.41]	0.247	0.25 [−1.01, 1.51]	0.689
MM injury	0.80 [−0.49, 2.08]	0.220	1.12 [0.08, 2.17]	**0.036**	0.96 [0.01, 1.91]	**0.048**	0.08 [−1.26, 1.42]	0.903
LTS	0.03 [−0.21, 0.27]	0.805	0.01 [−0.16, 0.19]	0.871	0.00 [−0.17, 0.17]	0.983	0.05 [−0.19, 0.30]	0.654
MTS	−0.17 [−0.46, 0.13]	0.260	−0.14 [−0.37, 0.08]	0.200	−0.13 [−0.33, 0.08]	0.232	−0.12 [−0.44, 0.20]	0.454

Abbreviations: ALL, anterolateral ligament; CI, confidence interval; G‐PATS, global passive anterior tibial subluxation; LM, lateral meniscus; L‐PATS, lateral passive anterior tibial subluxation; LTS, lateral tibial slope; MM, medial meniscus; M‐PATS, medial passive anterior tibial subluxation; MTS, medial tibial slope; PATS, passive anterior tibial subluxation; R‐PATS, rotational passive anterior tibial subluxation; TFIS, time from injury to surgery.

^a^ Bold indicates significance.

### Exploratory analyses of the association between postoperative PATS and PROMs

At the 24‐month follow‐up, no significant associations were observed between the Lysholm score and L‐PATS (*β* = 0.08, *p* = 0.148), M‐PATS (*β* = 0.03, *p* = 0.352), G‐PATS (*β* = 0.05, *p* = 0.131) or R‐PATS (*β* = 0.04, *p* = 0.423). Similarly, no significant associations were observed between the IKDC score and L‐PATS (*β* = 0.00, *p* = 0.990), M‐PATS (*β* = 0.03, *p* = 0.390), G‐PATS (*β* = 0.02, *p* = 0.667) or R‐PATS (*β* = −0.03, *p* = 0.571) (Table 5).

**Table 5:** The association between postoperative PATS and PROMs at postoperative 24 months.

	Lysholm score		IKDC score	
	*β* [95% CI]	*p‐*Value	*β* [95% CI]	*p‐*Value
24‐month Postop. L‐PATS	0.08 [−0.03, 0.18]	0.148	0.00 [−0.11, 0.12]	0.990
24‐month Postop. M‐PATS	0.03 [−0.04, 0.10]	0.352	0.03 [−0.04, 0.11]	0.390
24‐month Postop. G‐PATS	0.05 [−0.02, 0.13]	0.131	0.02 [−0.06, 0.10]	0.667
24‐month Postop. R‐PATS	0.04 [−0.06, 0.15]	0.423	−0.03 [−0.15, 0.08]	0.571

Abbreviations: CI, confidence interval; G‐PATS, global passive anterior tibial subluxation; IKDC, International Knee Documentation Committee; L‐PATS, lateral passive anterior tibial subluxation; M‐PATS, medial passive anterior tibial subluxation; R‐PATS, rotational passive anterior tibial subluxation.

## DISCUSSION

The principal findings of the present study can be summarised as follows: (1) residual tibiofemoral malalignment characterised by increased L‐PATS, M‐PATS and G‐PATS can persist following ACLR, and (2) excessive preoperative PATS, ALL abnormality and medial meniscal injury were associated with increased postoperative PATS.

The concept of irreducible anterior tibial subluxation was introduced by Almekinders et al. [1, 2], highlighting the abnormal tibiofemoral alignment visualised on stress radiographs in ACL‐injured knees despite undergoing ACLR. Similarly, Tagesson et al. reported no difference in static anterior tibial translation before and after ACLR in a 5‐year follow‐up study of 10 patients [31]. In a recent study, Karatekin et al. observed no reduction in L‐PATS measured at 6 months postoperatively compared with the preoperative status, suggesting that ACLR might not be able to restore native tibiofemoral alignment [13]. On the contrary, immediate MRI examinations after ACLR demonstrated that PATS could be reduced to −3.1 ± 2.5 mm, indicating a posteriorly over‐reduced position of the tibia [33]. Moreover, Muller et al. found a remaining difference of merely 1.0 ± 2.1 mm from the healthy limb in PATS with a minimum follow‐up of 4 months after anatomic ACLR [20], concluding that ACLR could approach native tibiofemoral relationships. The discrepant results among previous studies highlight the necessity of longitudinally tracking of postoperative PATS, as PATS only characterises the tibiofemoral relationship at the time of MRI examination and is not supposed to be static over time [6, 18].

The present study is the first to systematically examine the postoperative changes in PATS at multiple time points. Despite clinically successful ACLR characterised by satisfactory clinical outcomes, serial MRI revealed significant increases in PATS in both lateral and medial compartments postoperatively, which became the most prominent at 12 months and remained stable thereafter. The observed changes in PATS provide a plausible explanation to reconcile the seemingly contradictory findings from previous studies, confirming the presence of tibiofemoral malalignment in successfully reconstructed knees [1, 2, 31, 37]. Combined with the previously reported immediate decrease in PATS postoperatively [20, 33], it is possible that PATS undergoes a temporal reduction after an isolated ACLR and increases thereafter. However, immediate changes in PATS were not assessed in the present study due to the lack of immediate postoperative MRI evaluations.

Surprisingly, residual PATS persists following ACLR despite graft integrity being confirmed by physical examination and MRI evaluation. However, these findings corroborate previous evidence showing that the tibiofemoral malalignment measured by radiograph is not necessarily reduced by the surgery despite the improvement in stability measured by KT‐1000 [1, 2, 25]. This suggests that the underlying mechanisms of PATS may differ from the anterior tibial translation that occurs during a dynamic laxity test and [5, 25], therefore, may be overlooked in the routine evaluation during clinical practice. Although abnormal tibiofemoral alignment characterised by excessive PATS does not directly imply pathologic knee kinematics during motion [31], the residual tibiofemoral malalignment warrants further investigation. It has been suggested that increased PATS is associated with knee instability [15, 17, 36], inferior subjective outcomes [28] and graft failure [6, 19, 32]. Moreover, the increased anterior tibial translation and internal rotation could alter joint loading patterns and lead to early onset and progression of knee osteoarthritis [30], which still prevails in successfully reconstructed knees at high incidence rates [3]. Although residual PATS did not correlate with short‐term clinical scores in this cohort, its persistence suggests that isolated ACLR may not fully restore native tibiofemoral alignment, warranting further investigation into its long‐term impact on joint health.

Several hypotheses have been formulated regarding the underlying causes of residual PATS, including the fibrotic response of the posterior cruciate ligament secondary to the intraoperative violation [2], and the imbalance between extensor and flexor muscle strength following hamstring graft harvesting [13]. More recently, increasing evidence has suggested a multifactorial etiology for PATS, which is not only related to ACL deficiency but also affected by injuries to secondary stabilisers, including the anterolateral complex, menisci and joint capsule [19, 23, 38, 39]. Therefore, it was hypothesised that surgery limited to ACLR might not be able to fully restore the native knee kinematics in certain situations [13, 26, 28], especially in patients demonstrating inferior tibiofemoral alignment preoperatively [4, 5, 12]. In agreement with previous evidence, the present study demonstrated strong correlations between preoperative and postoperative values for L‐PATS, M‐PATS, G‐PATS and R‐PATS (all *p* < 0.001). This finding justifies the concern that excessive preoperative PATS predisposes patients to a higher risk of residual tibiofemoral malalignment [13, 28, 37]. Meticulous evaluation to identify the contributing factors and, where possible, address these factors via individualised surgical plans might help improve the clinical outcomes.

The contribution of secondary stabilisers in controlling tibiofemoral alignment has been validated in multiple clinical and biomechanical studies. The ALL has been reported as a crucial component of the anterolateral complex, which functions as a restraint to anterior tibial translation and internal rotation [14]. Occurring concomitantly with more than half of ACL injuries [21], ALL injuries are associated with high‐grade knee laxity in both acute and chronic cases [36], as well as excessive PATS in ACL‐deficient knees [21, 39]. Findings of the present study indicate that MRI‐detected ALL abnormalities are predictive of a mean increase in L‐PATS of 1.85 mm and G‐PATS of 1.31 mm at 24 months postoperatively, which is in line with a previous study reporting a 1.8 mm increase in L‐PATS with ALL injuries [39]. The menisci, another component of the secondary stabilisers, also aid in the control of anterior tibial translation [18]. Medial meniscal injury has been found to generate anterior tibial translation and anteroposterior laxity in both ACL‐deficient and reconstructed knees [4, 5, 21]. In agreement with previous evidence, the present study demonstrates medial meniscal injury is associated with a 1.12 mm increase in M‐PATS and a 0.96 mm increase in G‐PATS at 24 months postoperatively. Together, these results highlight the importance of secondary stabilisers as restraints on PATS. Deficiencies of these secondary stabilisers, caused either by acute insult during the primary injury or by the cumulative damage due to the unaddressed instability could potentially result in the residual tibiofemoral malalignment [18, 19, 26], as was indicated by the gradual increase in PATS among healthy controls, primary ACL deficiencies and single and recurrent graft failures [6, 19, 32].

Efforts have been made to correct excessive PATS and diminish related detrimental effects. Double‐bundle techniques, ALL reconstruction and slope‐reducing tibial osteotomy have been proposed and implemented as interventions to help reduce PATS, showing improved instrumented stability and patient‐reported outcomes [16, 29, 35]. Although a definite indication for these interventions cannot be established from the present study, the findings suggest that patients presenting with excessive preoperative PATS are at a higher risk of residual tibiofemoral malalignment. Individualised surgical plans aiming to provide better restraints on anteroposterior and rotational stability should be considered in this population [28]. Further investigations on the prognostic value of PATS in ACL‐reconstructed patients, especially regarding its relationship with knee stability, graft survivorship and osteoarthritis progression, would help determine the necessity and indications for these interventions.

## LIMITATIONS

Several limitations of the present study need to be acknowledged. First, only anatomic ACLRs using hamstring tendon autografts were analysed, which could limit the generalisability of the findings. Second, PATS measured by MRI in a supine position allowed for a precise and quantitative evaluation of the tibiofemoral relationship in both compartments individually. However, this static measurement might not reflect the actual knee kinematics during weight‐bearing. Third, it was not possible to determine the extent to which postoperative PATS deviated from normal values in the absence of healthy individuals or contralateral knees as controls. However, a self‐controlled design was sufficient to illustrate residual PATS. Fourth, the limited sample size made it infeasible to perform subgroup analyses or account for additional potential confounders. Fifth, the pivot‐shift test and instrumented laxity test were not performed in the present study. Therefore, no conclusion could be drawn on the relationship between PATS and knee stability given the potential bias from the subjective nature of manual examinations.

## CONCLUSIONS

Residual tibiofemoral malalignment characterised by increased L‐PATS, M‐PATS and G‐PATS persists following ACLR using hamstring tendon autografts. Excessive preoperative PATS, ALL abnormality and medial meniscal injury are associated with increased postoperative PATS.

## AUTHOR CONTRIBUTIONS

**Yi‐tian Gao**: Conceptualisation; draft writing. **Wei‐li Shi**: Data collection; draft writing. **Zhi‐yu Zhang**: Data collection; statistical analysis. **Wen‐bin Bai**: Data collection; statistical analysis. **Zi‐hao Zhou**: Data collection; statistical analysis. **Yu‐ping Yang**: Surgical data provision; draft revision. **Yong Ma**: Data collection; draft revision. **Xi Gong**: Surgical data provision; draft revision. **Cheng Wang**: Data collection; surgical data provision. **Jian‐quan Wang**: Conceptualisation; surgical data provision; supervision.

## CONFLICT OF INTEREST STATEMENT

The authors have declared that there are no conflicts of interest in the authorship and publication of this contribution.

## ETHICS STATEMENT

This study was approved by the ethics committee of Peking University Third Hospital (Project Number: M2020384). Informed consent was obtained from all included patients.

## Data Availability

The data that support the findings of this study are available from the corresponding author upon reasonable request.
